# The impact of a standardized incident reporting system in the perioperative setting: a single center experience on 2,563 ‘near-misses’ and adverse events

**DOI:** 10.1186/s13037-014-0046-1

**Published:** 2014-12-10

**Authors:** Anita J Heideveld-Chevalking, Hiske Calsbeek, Johan Damen, Hein Gooszen, André P Wolff

**Affiliations:** Department of Operating Theatres, Radboud University Medical Center, Geert Grooteplein-Zuid 10, Internal postal code 738, 6525 GA Nijmegen, The Netherlands; Department of IQ Healthcare, Radboud University Medical Center, Nijmegen, The Netherlands; Department of Anesthesiology, Radboud University Medical Center, Nijmegen, The Netherlands

**Keywords:** Hospital incident reporting, Guideline adherence, Patient safety, Perioperative care, Quality improvement

## Abstract

**Background:**

The reduction of perioperative harm is a major priority of in-hospital health care and the reporting of incidents and their causes is an important source of information to improve perioperative patient safety. We explored the number, nature and causes of voluntarily reported perioperative incidents in order to highlight the areas where further efforts are required to improve patient safety.

**Methods:**

Data from the Hospital Incident Management System (HIMS), entered in the period from July 2009 to July 2012, were analyzed in a Dutch university hospital. Employees in the perioperatve field filled out a semi-structured digital form of the reporting system. The risk classification of the reported adverse events and ‘near misses’ was based on the estimated patient consequences and the risk of recurrence, according to national guidelines. Predefined reported incident causes were categorized as human, organizational, technical and patient related.

**Results:**

In total, 2,563 incidents (1,300 adverse events and 1,263 ‘near-miss’ events) were reported during 67,360 operations. Reporters were anesthesia, operating room and recovery nurses (37%), ward nurses (31%), physicians (17%), administrative personnel (5%), others (6%) and unmentioned (3%). A total of 414 (16%) adverse events had patient consequences (which affected 0,6% of all surgery patients), estimated as catastrophic in 2, very serious in 34, serious in 105, and marginally serious in 273 cases. Shortcomings in communication was the most frequent reported type of incidents. Non-compliance with Standard Operating Procedures (SOPs: instructions, regulations, protocols and guidelines) was reported with 877 (34%) of incident reports. In total, 1,194 (27%) voluntarily reported causes were SOP-related, mainly human-based (79%) and partially organization-based (21%). SOP-related incidents were not associated with more patient consequences than other voluntarily reported incidents. Furthermore ‘mistake or forgotten’ (15%) and ‘communication problems’ (11%) were frequently reported causes of incidents.

**Conclusions:**

The analysis of voluntarily reported perioperative incidents identified an association between perioperative patient safety problems and human failure, such as SOP non-compliance, mistakes, forgetting, and shortcomings in communication. The data suggest that professionals themselves indicate that SOP compliance in combination with other human failures provide room for improvement.

## Background

Patient safety is a global public health issue receiving rapidly increasing attention. Patient safety is the (near) absence of (the chance of) avoidable harm inflicted on the patient through the actions and/or negligence of employees or through flaws in the healthcare system [1]. Numerous medical record studies have shown that unsafe care may result in adverse events (AEs) leading to harm in 3-17% of hospital patients [2]. An AE is “an unintended injury or complication resulting in prolonged length of hospital stay, disability at the time of discharge or death caused by health care management and not by the patients’ underlying disease” [3]. A total of 51-77% of AEs in hospitals are related to perioperative care [2]. A systematic review reveals that 14% of perioperative patients experience some form of AEs, that 38% of these AEs are preventable and that 4% of patients experiencing AEs have fatal outcomes [4]. Preventable AEs are the result of care that falls below current professional standards and the expected performance of practitioners or care systems [1,2].

To improve patient safety and reduce adverse events, a national safety management system has been implemented in Dutch hospitals (‘Veiligheidsmanagementsysteem = VMS’) between 2008–2012. VMS focuses on the management of risks (the prevention of patient harm and its possible consequences) and is described in the ‘Netherlands Technical Agreement’ (NTA) [5]. The starting point for this NTA was the report “Here you’ll work safely, or you won’t work here” [6]. VMS consists of systems for risk identification, risk analysis, risk evaluation, incident reporting, incident analysis, and managing recommendations and improvement measures.

The operating department (including the clinical operating and recovery rooms) of the Radboud university medical center introduced the Hospital Incident Management System (HIMS) in July 2009. HIMS aimed at facilitating the voluntarily and confidentially reporting of perioperative incidents. After using HIMS for three years, we set out to analyze whether there are specific types or patterns of the reported information and to detect the areas where further efforts are required to improve perioperative patient safety. Specifically, we wished to investigate the number and characteristics of the voluntarily reported perioperative incidents. From the start of recording we noticed that Standard Operating Procedure (SOP) non-compliance was a prominent reported cause of incidents.

Since humans are fallible, systems must be designed to prevent humans from making errors [7,8]. Standardisation, the use of guidelines and protocols, is generally considered to improve perioperative safety. Preventable patient harm may be the result of guideline non-compliance but unfortunately there is hardly any literature about this problem in perioperative care. Dutch hospital record review studies suggest that at least 10-15% of AEs are directly or indirectly related to procedure non-compliance [9,10]. The compliance rate of the use of surgical safety checklists ranges from 12% to 100% (mean 75%) [11]. French research revealed that alarms are frequently ignored by the operating theatre staff [12]. English anesthesiologists witnessed that 22% of incidents during anaesthesia are related to protocol violations [13].

There is an association between undesirable perioperative events and subsequent critical perioperative outcomes [14]. Recent studies reveal also that better guideline compliance is associated with better perioperative outcomes [15,16]. Therefore, adherence to guidelines is an important target for safety improvement programs. This study was designed to provide data on the number, nature and causes of voluntarily reported perioperative incidents.

## Methods

This retrospective study is performed in the Radboud university medical center, which has 953 beds and 25 operating rooms (ORs). All data were obtained from HIMS, a database that is developed by the Patient Safety Company (http://www.patientsafety.com), licensed to the Radboud university medical center and used by the operating department since July 2009. The study period lasted from the 1^st^ of July 2009 to the 1^st^ of July 2012. Incidents in HIMS are reported as a) ‘adverse events’, i.e. any unintended or unexpected event which could have led or did lead to harm of one or more patients receiving hospital care and b) ‘near-miss events’, i.e. events or circumstances that nearly occurred but were prevented (through luck or intervention) and did not lead to patient harm. [1] Harm is defined as any injury to the patient which leads to an extension or increase in the treatment, to temporary or permanent physical, psychological and/or social functional loss, or to death [5]. The worsening of a patient’s condition as a result of the natural progression of an disease is not considered ‘harm’ [5].

**Figure 1:**
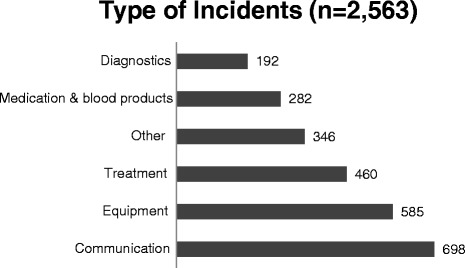
**Predefined types of voluntarily reported perioperative incidents.**

At the introduction of HIMS, hospital wide appointments were made for reporting of incidents, based on the national agreements [5]. All employees, including professionals working in the perioperative process were encouraged to report incidents in order to prevent them from happening again. They were requested to fill out a semi-structured digital form of the reporting system. At least the following had to be reported: date, time and location of the incident, a brief description of the event and the circumstances, the type of incident and possible causes, the potential patient impact and the estimated risk of recurrence of that incident, and the measures that may prevent the incident from repeating. The predefined categorization of incident types is illustrated in Figure 1. In Tables 1 and 2 an explanation of the used terms is given, in particular of the concepts estimated risk of recurrence (Table 1) and estimated consequences for the patient (Table 2). This categorization of patient consequences is based on national guidelines as described in the NTA [5]. Based on the estimated patient consequences and risk of recurrence, the incident is automatically classified into four risk categories (Table 3). This risk classification is the basis for actions to be taken by the Operating Room Incident Reporting Committee (ORIRC) as described in Table 4. According to the Dutch consensus classification reported incident causes were classified as human, organizational, technical, patient related and other [1,2].

**Table 1:** **Explanation of risk of recurrence**

**Classification of the estimated risk of recurrence:**	
Almost inevitable	It will probably happen again within a few hours or days
Probable	It will probably happen again within a week
Possible	It will probably happen again within a few weeks
Small	It will probably happen again within a few month
Very small	It will not happen more than once a year

The estimated risk of recurrence of the reported incidents.

**Table 2:** **Explanation of patient consequences**

**Classification of patient consequences:**	
Catastrophe	(Expected) death or (expected) severe permanent harm
Very serious	(Expected) permanent harm/major intervention like (re)operation and/or (expected) extended hospitalization or treatment > 7 days/delay of treatment causing severe risk of harm
Serious	(Expected) temporary harm and/or severe pain, for which medical treatment is needed and/or (expected) extended hospitalization or treatment > 3 days/delay of treatment causing risk of harm
Marginally serious	(Expected) minimal harm and/or pain, requiring minor treatment and/or (expected) extended hospitalization or treatment < 3 days/delay of treatment causing minimal risk of harm
None	No harm and no delay, or (expected) delay of treatment causing no harm

The estimated potential patient consequences of the reported incidents.

**Table 3:** **Explanation of risk matrix**

**Patient consequences**					
Catastrophe	Extreme	Extreme	Extreme	Extreme	Extreme
Very serious	High	High	High	High	High
Serious	High	High	High	Medium	Medium
Marginally serious	High	High	Medium	Medium	Low
None	Medium	Medium	Medium	Low	Low
**Risk of recurrence**	Almost Inevitable	Probable	Possible	Small	Very small

Risk matrix based on the estimated patient consequences and the estimated risk of recurrence of the incident.

**Table 4:** **Explanation of reporting requirments based on the classified risk**

Extreme risk incident	The reporter/ORIRC contacts the involved head of the department and checks whether the catastrophe is handled according to the standard procedures, meaning that the Board of Hospital Directors reports the catastrophe to the Health Care Inspectorate.
High risk incident	The ORIRC gathers further information, analyzes the incident, discusses the incident in a meeting, formulates conclusions and/or improvement actions.
Medium risk incident	The ORIRC gathers further information, discusses the incident in a meeting, formulates conclusions and/or improvement actions.
Low risk incident	The ORIRC formulates conclusions and/or improvement actions.

ORIRC = Operating Room Incident Reporting Committee.

Reporting requirements based on the classified risk.

The ORIRC consists of a surgeon, two anesthesiologists, a patient safety coordinator, a recovery room nurse, two operating room nurses, an anesthesia nurse, a logistic manager, a logistic employee, and a technician. The ORIRC reviews and discusses incidents weekly with priority to high risk events. Estimating the risk of recurrence and potential consequences for the patient at the time of reporting and before analysis of the incident may be difficult. Therefore, the ORIRC may adapt the risk score after analysis of the incident. Subsequently, the ORIRC undertakes actions, such as gathering additional information, cross-checking with the medical charts, and exploring key contributing factors through informal discussions with care providers, managers, and/or other expert(s), asking ‘why’ and ‘what’ influenced the occurrence of this (near)incident. Besides these analyzing actions, other activities of ORIRC are trend watching, advising to adjust products and procedures, providing feedback and spreading newsletters and safety alerts to all involved in perioperative care. After closing a report, all information is automatically anonymized. The information in HIMS is not used for blaming, shaming or (legal) actions against the reporting professional, but for improving the quality of patient care.

Descriptive statistics were used to describe the number, nature and causes of incidents. Chi-square tests with Yates’ correction and odds ratios (ORs) with 95% confidence limits (CL) were applied to test differences between physicians and non-physicians and between perioperative and hospital wide reporting behaviour. A p-value < 0.05 is considered to show statistical significance.

## Results

### Hospital-wide voluntarily reported incidents

Hospital-wide key figures over the period July 2009-July 2012 are presented in Table 5. The occurrence of voluntarily reported incidents associated with procedure non-compliance within the operating department was not significantly different from hospital wide reporting of non-compliance with SOPs (34% and 32,5% respectively (OR 1.087; 95% CL 0.998-1.184, p = 0.084)).

**Table 5:** **Hospital-wide key figures, July 2009-July 2012**

Total number of admissions	210,507
Total number of clinical operations	67,360
Total number of voluntarily reported incidents:	27,008
by physicians	2,937
by non-physicians	23,154
by reporters that did not mention their position	917
Number of voluntarily reported incidents per type:	
medication & blood-related	6,932
communication-related	6,053
diagnostics-related	4,183
treatment-related	3,751
equipment-related	2,421
other type-related	3,668
Total number of voluntarily reported causes (median 1; range 0–10 causes per incident)	48,055
Number of causes per category:	
mistake/forgotten	9,611
SOP not followed	6,535
other	31, 909
Total number of SOP-related causes:	10,543
SOP not followed	6,535
SOP not known	2,116
SOP not available, incomplete or unclear	1,684
SOP not accessible	208
Number of incidents with SOP cause	8,789

### Characteristics of the voluntarily reported perioperative incidents

Perioperatively 2,563 incidents were voluntarily reported in HIMS: 1,300 adverse events and 1,263 ‘near-miss’ events. The reporters were all professionals involved in perioperative patient care: anesthesia, operating room and recovery nurses (37%), ward nurses (31%), physicians (17%), administrative personnel (5%), and others (6%). Of 85 (3%) incidents the appointment of the reporter was not mentioned. Figure 1 provides an overview of the types of incidents; communication failures (27%) and equipment failures (23%) were the most frequently reported type of voluntarily reported perioperative incidents.

### Patient consequences and risk of recurrence of reported perioperative incidents

HIMS characterized 1,822 (71%) incidents as ‘medium risk incidents’ which is shown in Table 6. Table 7 presents a specification of the estimated patient consequences and risk of recurrence of incidents. Patient consequences were estimated to have been inflicted 414 (32%) of 1,300 adverse events: 273 events were considered to have caused minimal patient harm and/or pain, 105 temporary patient harm and/or severe pain, and 34 permanent patient harm and/or needing major intervention like (re)operation and/or extended hospitalization or treatment. Two catastrophic adverse events, with one mortality, were reported to both the hospital board and the Dutch Health Care Inspectorate. The risk of recurrence of the previously mentioned 414 adverse events was estimated as follows: possible recurrence within a day (n = 30), within a week (n = 71), within a few weeks (n = 220), within a few months (n = 70), and of 23 adverse events it was estimated that the event would occur not more frequently than once a year.

**Table 6:** **Risk classification of the 2,563 voluntarily reported perioperative incidents**

**Risk**	**Total incidents**	
	**n**	**%**
Extreme risk	3	(0.1%)
High risk	349	(13.6%)
Medium risk	1822	(71.1%)
Low risk	389	(15.2%)
**Total**	**2563**	

Risk classification of the 2,563 voluntarily reported perioperative incidents.

**Table 7:** **Estimated patient consequences and risk of recurrence of the 1,300 voluntarily reported perioperative adverse events**

**Estimated patient consequences**						**Total**
Catastrophe	0	0	0	1	1	**2**
Very serious	4	6	16	7	1	**34**
Serious	4	18	58	18	7	**105**
Marginally serious	22	47	146	44	14	**273**
None	134	228	335	138	51	**886**
**Total**	**164**	**299**	**555**	**208**	**74**	**1300**
**Estimated risk of recurrence**	Almost inevitable	Probable	Possible	Small	Negligible	

Estimated patient consequences and risk of recurrence of the 1,300 voluntarily reported perioperative adverse events.

The estimated patient consequences and risk of recurrence of reported perioperative ‘near-miss’ events are presented in Table 8. In total 233 (18%) of these 1263 ‘near-misses’ were estimated as potentially harmful, which was classified as marginally serious in 107 near-incidents, serious in 81, and very serious in 44. One potential catastrophic ‘near-miss’ event concerned mechanical problems with a part of the operating table and is described as an example in Table 9.

**Table 8:** **Estimated potential patient consequences and risk of recurrence of the 1,263 voluntarily reported perioperative ‘near-miss’ events**

**Patient consequences could have been**						**Total**
Catastrophe	0	0	1	0	0	**1**
Very serious	10	7	15	6	6	**44**
Serious	13	37	27	3	1	**81**
Marginally serious	19	26	49	11	2	**107**
None	205	207	434	138	46	**1030**
**Total**	**247**	**277**	**526**	**158**	**55**	**1263**
**Estimated risk of recurrence**	Almost inevitable	Probable	Possible	Small	Negligible	

Estimated potential patient consequences and risk of recurrence of the 1,263 voluntarily reported perioperative ‘near-miss’ events.

**Table 9:** **Examples of reported perioperative incidents in HIMS**

**Examples of reported perioperative incidents in HIMS**					
**HIMS risk classification**	**Description**	**Event type**	**Incident type**	**Function of reporter**	**Reported cause**
**Low risk**	*Two patients did not have correct marking signs although the surgeon had signed the checklist.*	Adverse event	Communication	Recovery nurse at the holding of the OR	SOP not followed
**Medium risk**	*During surgery the following happened:*	Adverse event	Other	Anesthetic nurse	- Other organization-related problem, namely: “delay because of shortage of staff”;
	- *30 minutes waiting because the patient arrived too late in the OR;*				
	- *For this operation there was no blood typing performed;*				- Human error or forgotten.
	- *During the time out it appeared that the right size implant was not available.*				
**High risk**	*Surgery was performed without recent available imaging. During surgery, it appeared that metastases were increased in size necessitating adjustment of the surgical procedure.*	Adverse event	Diagnostics	Radiologist	- SOP not known
					- SOP not available/incomplete/unclear
					- Incorrect performance
**Extreme risk**	*The headrest of the surgical table suddenly went loose, which could have caused the head of the patient to bend downwards uncontrollably but the head of the patient was stabilized in time by the anesthesiologist.*	‘Near-miss’ event	Equipment	OR nurse	- Broken material;
					- Wrong design;
					- Other human error, namely: “part of the table not correctly fixated”.

A total of 2,149 (84%) of the voluntarily reported perioperative incidents were considered to have caused no patient harm, or delay of treatment which caused no harm, as shown in the Tables 7 and 8. The risk of recurrence in 381 of these 2,149 incidents were estimated to reoccur within a day, 505 within a week, 861 within a few weeks, 296 within a few months, and of 106 incidents it was estimated that the event would occur no more than once a year.

### Reported causes

Table 10 shows the reported predefined causes of perioperative incidents and their classification. A total of 4,346 causes were reported (median 1, range 0–10 per incident). In total 2,966 (68%) incident causes were related to human factors, 1,004 (23%) to organizational factors, 89 (2%) to technical failure, 128 (3%) were patient-related and 159 (4%) were related to other factors. Most frequently reported causes were SOP not followed (16,2%), human mistake or having forgotten (15,4%) and communication problems (11,5%). In total 1194 (27,5%) SOP related causes were noted, as summarized in Table 10 part b.

**Table 10:** **Causes of perioperative ‘near-misses’ and adverse events**

**A: Reported causes (n = 4,346) of the perioperative incidents (n = 2,563)**		
**HIMS predefined causes**	**N**	**%**
**Human**		
SOP not followed	702	16.2%
Mistake/forgotten	669	15.4%
Communication problem	498	11.5%
Other human acting, namely^*)^	449	10.3%
SOP not known	245	5.6%
Professional not capable for task	161	3.7%
Distracted	105	2.4%
Unqualified or incorrect performance	77	1.8%
Incorrect use	40	0.9%
Wrong record filing	20	0.5%
**Total human**	**2,966**	**68.2%**
**Organizational**		
Other organizational, namely^*)^	315	7.2%
SOP not available, incomplete, or unclear	217	5.0%
Culture at workplace	114	2.6%
High workload	115	2.6%
Equipment/supply related, namely^*)^	67	1.5%
Inadequately trained professional	67	1.5%
Medical devices not available	62	1.4%
SOP not accessible	30	0.7%
Unclear instructions	17	0.4%
**Total organizational**	**1,004**	**23.1%**
**Technical**		
Broken material	61	1.4%
Wrong design	28	0.6%
**Total technical**	**89**	**2.0%**
**Patient-related**		
Other patient related, namely^*)^	91	2.1%
Patient condition	19	0.4%
Patient behaviour	18	0.4%
**Total patient-related**	**128**	**2.9%**
**Other, namely** ^*)^	**159**	**3.7%**
**Total**	**4,346**	**100%**
**B: Summary of SOP releated causes**		
SOP not followed	702	16.2%
SOP not known	245	5.6%
SOP not available, incomplete, or unclear	217	5.0%
SOP not accessible	30	0.7%
**Total of SOP related causes**	**1,194**	**27,5%**

^*)^Further described by the reporter in open text field.

SOP = Standard Operative Procedure, including instructions, regulations, protocols, and guidelines.

### Reported causes of procedure non-compliance

A more detailed analysis of the 1,194 SOP related causes showed that 79% was related to human failure: a SOP was known but not followed in 702 cases and not known in 245 cases. Organization related factors were: unavailable, inaccessible, incomplete and unclear SOPs in 247 (21%) of cases. Procedure non-compliance was reported with 877 (34%) of incidents; 648 SOP causes were reported with 471 adverse events, and 546 with 406 ‘near-miss’events.

Table 11 summarize the patient consequences and risk of recurrence of SOP associated perioperative incidents. In 151 of the 877 SOP related incidents, the events were associated with patient harm, while 263 of 1,686 non SOP related incidents were associated with patient harm (OR 1.13 (95% CL 0.90-1.40), p = 0.291).

**Table 11:** **Patient consequences and risk of recurrence of SOP related incidents**

**A. Reported SOP-related perioperative adverse events and ‘near-miss’events (n = 877)**						
**Estimated Patient consequences**						**Total**
Catastrophe	0	0	0	0	0	**0**
Very serious	6	2	4	6	1	**19**
Serious	6	12	39	4	0	**61**
Marginally serious	15	27	79	17	4	**142**
None	139	164	254	72	26	**655**
**Total**	**166**	**205**	**376**	**99**	**31**	**877**
**Estimated risk of recurrence**	Almost inevitable	Probable	Possible	Small	Negligible	
**B. Reported SOP-related perioperative adverse events (n = 471)**						
Catastrophe	0	0	0	0	0	**0**
Very serious	1	1	4	3	1	**10**
Serious	2	9	25	3	0	**39**
Marginally serious	8	16	62	13	3	**102**
None	59	91	117	39	14	**320**
**Total**	**70**	**117**	**208**	**58**	**18**	**471**
**Estimated risk of recurrence**	Almost inevitable	Probable	Possible	Small	Negligible	

Most SOP related causes (n = 992) were reported by non-physicians, 170 by physicians and 32 by reporters who did not mention their function in the organization. Non-physicians reported less frequently SOP causes of incidents than physicians (52% versus 68%, p < 0.0001), but non-physicians reported more often SOP causes of near-incidents (48% versus 32%, p < 0.0001). There was no statistically significant difference between both groups in reporting human or organization related factors of SOP non-compliance. Both groups most frequently reported SOP causes with incidents that were classified as medium risk incidents.

To illustrate the characteristics of voluntarily reported perioperative incidents, examples are described in Table 9.

## Discussion

We studied the characteristics of voluntarily reported perioperative incidents at our operating department in the period July 2009-July 2012. Reported incidents included ‘near-miss’ events as they can be seen as ‘free lessons to be learned’ [17]. Overall, 67,360 operations were performed in the three-year study period and 2,563 perioperative incidents were voluntarily reported. Most (84%) of these incidents were not considered as potentially harmful for patients, but 16% were, which comprised 0.6% of all surgery patients. SOP non-compliance, shortcomings in communication, and mistakes or just ‘having forgotten’, appeared to be frequently reported incident causes. About one third of the reported perioperative incidents was associated with SOP non-compliance; 79% of these SOP-related causes were related to human failure. The SOP-related incidents were not associated with more patient consequences than other incidents. It is noteworthy that the percentage voluntarily reported incidents associated with SOP non-compliance within the operating department did not differ from the hospital-wide incidence.

There is increasing information that better guideline compliance is associated with improved perioperative outcomes [15,16]. A study on the implementation of a bundle of care to reduce postoperative surgical site infections showed that increased bundle compliance from 10% to 60% was associated with a significant 36% reduction in infection rate [16]. Another study in a Dutch university hospital on the effect of the use of a perioperative safety checklist showed a significant 56% reduction of the in-hospital 30-day mortality after surgery in patients with completed checklists whereas the mortality rate remained unchanged in patients with partially completed or non-completed checklists [15]. The results of our study clearly show that a thorough analysis of the barriers that hinder adequate communication and compliance with SOPs is essential to further improve perioperative safety [18–20].

Incident reporting and investigation was first used in the 1940s to improve safety and performance of military aviation [21] and some decades later incident reporting was introduced in anesthesiology [22]. Meanwhile, incident reporting has become a widely recommended method to gather information about AEs in hospital care [18] and several methods have been developed [13,23–25]. Much information about AEs in hospitals comes from the retrospective study of medical records [2,4,26,27]. However, retrospective record review has the well-known disadvantage that AEs may not have been identified or reported because they were not recorded in the medical file. These retrospective studies also rarely give information about ‘near-miss’events. Patient’s information is also a reliable source for the identification of care-related AEs [28]. Monitoring the performance of professionals by trained observers may provide the most reliable picture of perioperative practice [13,29]. However, these studies may underreport the actual rate of AEs because the researchers only observed during daytime of weekdays [29]. Smith at al. witnessed that around 20% of the incidents reported were the result of violation from existing protocols [13].

To further improve patient safety it is essential not only to get insight into the number, type, risk and causes of voluntarily reported incidents, but also to reach agreement on definitions and reproducible ways of reporting and scoring. The Netherlands Technical Agreement aims to contribute to the uniformity of the safety management system in hospitals and to create openness about patient safety towards patients and public. In order to improve the effectiveness of the national incident reporting system, we suggest further standardization of incident reporting data, specifically of the categorization of the types and causes of reported incidents. This has the potential to provide us with a system to compare hospital performance and to benchmark performance using a validated system.

The five basic elements essential for the successful translation from incident reporting to learning are 1) an open reporting culture allowing independent non-punitive data input, 2) the opportunity to freely narrate one’s own version of the event, 3) an analysis that turns the incident report into a lesson, 4) adequate feedback [23], and 5) definitions clear enough to be used in other centres in order to be able to compare hospital performances. Investigating and analyzing incidents requires optimal engagement of physicians to get insight into the root cause and prevention of these events [23]. The use of a standardized framework for analysis of events has been recommended and introduced in several centers around the world including our center [23,30]. A positive safety culture is associated with increased willingness to report errors [31,32], with increased compliance with SOPs [33] and with fewer AEs [34]. Apparently professionals at our department felt safe enough to report communication failures and non-compliance with SOPs. They knew that the information will not be used to blame or undertake actions against the reporter and that after analysis of an incident the information was anonymized to be used for training purposes and improving the quality of care, if deemed useful for this purpose.

A major weakness of incident reporting in all studies is under-reporting. Incident reporting may capture only 4% to 50% of AEs [25,27,32–38]. The reasons for not reporting are numerous: clinical factors (e.g. emergency scenario), time constraints, unfamiliarity with the system, problems with the definitions of what constitutes a reportable incident or near-incident, lack of a hospital policy of ‘no disciplinary action’ on incident reporting, lack of anonymity, lack of feedback and confusing aims of the reporting system as such [13,18]. However, the best policy to improve reporting behaviour is currently unknown [39]. Unfortunately, because of the risk of under-reporting, voluntary incident reporting is not a reliable instrument to monitor performance in e.g. the plan-do-study-act cycle. However, it may be used as qualitative monitor to identify areas that require further efforts to improve perioperative patient safety.

### Strength and limitations of the study

We have used voluntarily reporting of incidents as an important source of information and hypothesized that this would make us capable to contribute to understanding and identifying clues for improving perioperative safety [23,40]. The present study highlights the areas where further efforts are required to improve patient safety. Because of the likelihood of under-reporting, our dataset cannot be considered a reproduction of all actually occurred incidents. A second point of attention is the fact that a substantial part of perioperative care is delivered outside the operating department. Therefore, our results, collected in the operating department, do not represent the full scope of perioperative incidents, as an unknown proportion will have been reported to other incident reporting committees within the hospital. Finally, this single center study may only reflect the culture and customs of our hospital. Since non-compliance with guidelines is a well-known phenomenon, our results are most likely not specific for our hospital. However, there is no information about the contribution of non-compliance with SOPs as cause of perioperative incidents in other hospitals.

## Conclusions

Voluntarily incident reporting provide important and detailed information about perioperative patient safety problems. The most important finding of the present study is that professionals themselves report non-compliance with SOPs associated with human failure is an important area for improvement. Furthermore shortcomings in communication, mistakes, and forgetting were identified as important targets for improvement to reduce perioperative incidents in our hospital. This finding requires acknowledgement of the risk of human attitude, behaviour and failure. Moreover these findings challenge for the development of tools to improve guideline adherence and effective communication, in order to improve perioperative patient safety.
